# Improved glucose tolerance with DPPIV inhibition requires β‐cell SENP1 amplification of glucose‐stimulated insulin secretion

**DOI:** 10.14814/phy2.14420

**Published:** 2020-04-27

**Authors:** Mourad Ferdaoussi, Nancy Smith, Haopeng Lin, Austin Bautista, Aliya F. Spigelman, James Lyon, XiaoQing Dai, Jocelyn E. Manning Fox, Patrick E. MacDonald

**Affiliations:** ^1^ Department of Pharmacology and Alberta Diabetes Institute University of Alberta Edmonton AB Canada

## Abstract

Pancreatic islet insulin secretion is amplified by both metabolic and receptor‐mediated signaling pathways. The incretin‐mimetic and DPPIV inhibitor anti‐diabetic drugs increase insulin secretion, but in humans this can be variable both in vitro and in vivo. We examined the correlation of GLP‐1 induced insulin secretion from human islets with key donor characteristics, glucose‐responsiveness, and the ability of glucose to augment exocytosis in β‐cells. No clear correlation was observed between several donor or organ processing parameters and the ability of Exendin 4 to enhance insulin secretion. The ability of glucose to facilitate β‐cell exocytosis was, however, significantly correlated with responses to Exendin 4. We therefore studied the effect of impaired glucose‐dependent amplification of insulin exocytosis on responses to DPPIV inhibition (MK‐0626) in vivo using pancreas and β‐cell specific sentrin‐specific protease‐1 (SENP1) mice which exhibit impaired metabolic amplification of insulin exocytosis. Glucose tolerance was improved, and plasma insulin was increased, following either acute or 4 week treatment of wild‐type (βSENP1^+/+^) mice with MK‐0626. This DPPIV inhibitor was ineffective in βSENP1^+/−^ or βSENP1^−^^/^^−^ mice. Finally, we confirm impaired exocytotic responses of β‐cells and reduced insulin secretion from islets of βSENP1^−^^/^^−^ mice and show that the ability of Exendin 4 to enhance exocytosis is lost in these cells. Thus, an impaired ability of glucose to amplify insulin exocytosis results in a deficient effect of DPPIV inhibition to improve in vivo insulin responses and glucose tolerance.

## Graphical abstract

In vivo responses to incretin mimetics and DPPIV inhibitors is variable. We asked whether in vitro insulin responses of islets also varied, and whether this was related to glucose responsiveness. Finally, in vivo glucose lowering actions of DPPIV inhibition was found to require the glucose‐amplifying effects of the deSUMOylating enzyme SENP1.

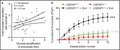

## 1 INTRODUCTION

In humans, the ability of glucose to stimulate insulin secretion from pancreatic islets is heterogeneous (Kayton et al., 2015; Lyon et al., 2016), as are responses to activation of the incretin receptors in vitro (Kolic, Spigelman, Smith, Manning Fox, and MacDonald, 2014) and to incretin mimetics (Jones, Shields, Hyde, Henley, and Hattersley, 2014) or DPPIV inhibitors (Kanamori and Matsuba, 2013) in vivo. We know very little about the underlying mechanism(s) responsible for these heterogenous responses, or whether variation in glucose‐regulated insulin secretory capacity can itself explain the responses to the incretin‐based therapies. It is likely that both genetic and environmental factors contribute to the heterogenous secretory responses of human islets to glucose and incretin‐based therapies (Franks and McCarthy, 2016).

Glucose‐metabolism and incretin‐signaling pathways both exert significant ‘amplifying’ actions on insulin secretion (Tudurí, López, Diéguez, Nadal, and Nogueiras, 2016; Ferdaoussi and MacDonald, 2017) that serve to enhance the exocytosis of insulin, independently from the enhancement of electrical activity and intracellular Ca^2+^. In addition to the ATP‐dependent depolarization, action potential firing, and increases in intracellular Ca^2+^ stimulated by glucose (collectively called the ‘triggering’ pathway), a secondary pathway which may be mediated by multiple possible metabolic signals (Prentki, Matschinsky, and Madiraju, 2013) acts to facilitate exocytotic responses to the increased Ca^2+^. In fact, these ‘metabolic amplification’ pathways may act early to set the amplitude of glucose‐induced secretory responses – and reduced efficacy of this pathway may contribute to impaired insulin secretion in type 2 diabetes (T2D) (Grespan et al., 2018). One important pathway that contributes to the metabolic amplification of insulin secretion links the mitochondrial export of (iso)citrate and cytosolic generation of NADPH (Joseph et al., 2006; Ronnebaum et al., 2006) which facilitates insulin exocytosis (Ivarsson et al., 2005; Reinbothe et al., 2009) via the deSUMOylating enzyme sentrin‐specific protease‐1 (SENP1) (Ferdaoussi et al., 2015) acting on proteins at the exocytotic site (Dai et al., 2011; Ferdaoussi et al., 2017).

The ‘receptor‐mediated amplification’ of insulin secretion by incretin hormones is mediated by cAMP‐independent pathways working through PI3 kinase to promote actin reorganization and insulin granule trafficking (Kolic et al., 2014; Kolic and MacDonald, 2015), and cAMP‐dependent pathways acting through PKA and Epac2A to phosphorylate exocytotic proteins and control insulin granule priming (Song et al., 2011; Wu et al., 2015; Alenkvist, Gandasi, Barg, and Tengholm, 2017). Although incretin‐induced insulin secretion is well‐known to be glucose dependent, the exact interaction between pathways controlling glucose‐dependent and incretin‐dependent facilitation of insulin secretion is unclear. We therefore sought to investigate the variability of human islet insulin responses to the glucagon‐like peptide‐1 (GLP‐1) receptor agonist Exendin 4, and the correlation of these responses to donor characteristics and/or the ability of glucose to amplify insulin granule exocytosis. We find that heterogeneity in human islet insulin responses to Exendin 4 are not easily explained by donor characteristics that include age, gender, or BMI; or characteristics of the islet isolation procedure itself (e.g., cold ischemia time, digestion time, culture time). Rather, Exendin 4 induced insulin secretion correlated with the ability of glucose to amplify insulin exocytotic responses – suggesting that glucose‐dependent effects on the exocytotic machinery could determine the extent to which incretin signaling is able to promote insulin secretion. We then tested the contribution of metabolic amplification to in vivo responsiveness to DPPIV inhibition using β‐cell‐specific SENP1 knockout (βSENP1‐KO) mice with a known deficiency in the glucose‐dependent amplification of insulin secretion. Homozygous and heterozygous mice deficient in β‐cell SENP1 showed impaired responsiveness in circulating insulin and reduced improvement in oral glucose tolerance to DPPIV inhibitor administered either acutely or over a period of several weeks. Thus, we demonstrate the correlation of human islet responses to Exendin 4 with a pathway mediating the metabolic‐amplification of insulin exocytosis in β‐cells. In mice, loss of this pathway blunts the improvements in glucose tolerance conferred by treatment with DPPIV inhibitors.

## 2 METHODS

### 2.1 Human pancreatic islets and static insulin secretion assays

Human islets were isolated in‐house (http://www.bcell.org/isletcore.html) from pancreas of cadaveric organ donors by standard collagenase perfusion, mechanical digestion, and density centrifugation (Kin and Shapiro, 2010; Lyon et al., 2016). A total of 86 donors were examined, and detailed information and β‐cell function data for each donor is provided in Table S1. The presence of type 2 diabetes was determined either by clinical information provided at the time of organ procurement, or by HbA1c > 6.5%.

Islets were handpicked and cultured overnight in DMEM (Gibco 11,885) supplemented with 10% FBS (Gibco 12483–020) and 1% Pencillin Streptomycin (Gibco 15140–122) at 37°C, 5% CO_2_. Islets were preincubated for 2 hr with 1 mM glucose KRB (115 mM NaCl, 5 mM KCl, 24 mM NaHCO_3_, 2.5 mM CaCl_2_, 1 mM MgCl_2_, 10 mM HEPES, 0.1% bovine serum albumin, pH 7.4). In triplicates, 15 islets were picked into tubes and incubated for 1 hr with 1 mM glucose KRB with or without 10 nM Exendin 4 (Sigma). Supernatant was collected and replaced with 10 mM glucose KRB with or without 10 nM Exendin 4 (Sigma) for 1 hr. Supernatant is collected and replaced with acid ethanol to extract content. All samples are stored at −20°C and assayed using electrochemiluminescence (Alpco, Salem, NH or Meso Scale Discovery).

### 2.2 Patch‐clamp electrophysiology

For electrophysiological assay of single‐cell exocytosis, islets were hand‐picked and dispersed to single cells by gentle shaking in Ca^2+^‐free buffer (Ferdaoussi et al., 2015). Patch‐clamp was performed using pipettes pulled from thin‐walled borosilicate tubes coated with Sylgard and having a resistance of 3–5 megaOhm after fire polishing. The intracellular and extracellular solutions, and protocol for assay of exocytotic responses were as described previously (Kolic et al., 2014).

### 2.3 β‐cell specific SENP1 knockout mice

SENP1‐floxed mice on a C57/B6 background were generated as previously described (Ferdaoussi et al., 2015) and crossed with the INS1‐Cre mice on a mix C57/B6 and SV129 background (Thorens et al., 2015) to generate wild‐type controls (+/+ Cre+; βSENP1^+/+^), heterozygotes (+/flox Cre+; βSENP1^+/−^), and knockouts (flox/flox Cre+; βSENP1^−^^/^^−^). Experiments were performed on male littermates, 10 weeks of age. Ear notches were used to determine mice genotypes as previously described (Ferdaoussi et al., 2015). Loss of SENP1 expression was confirmed by western blotting of islet lysates and nested qPCR for the presence of SENP1 transcript containing the targeted deletion (not shown). In the chronic study, mice were daily given DPPIV inhibitor (MK‐0626; 3 mg/Kg) or vehicle control (H_2_O) by gavage for four weeks. The oral glucose tolerance tests (OGTTs) was performed 20–24 hr after the last DPPIV inhibitor intake. The daily intake was discontinued for 3 weeks to washout the effect of DPPIV inhibitor. Then the mice were given DPPIV inhibitor (MK‐0626; 3 mg/Kg) or vehicle control by gavage 1 hr prior the OGTT to assess the acute effect of DPPIV inhibitor.

Prior to oral glucose tolerance tests (OGTTs), mice were given DPPIV inhibitor (MK‐0626; 3 mg/Kg) or vehicle control by gavage either 1 hr prior to the OGTT (acutely) or daily for 4 weeks (chronic). In the chronic study, mice were not given DPPIV inhibitor for 20–24 hr prior to OGTT.

### 2.4 Oral glucose tolerance tests

Mice were fasted for 4–5 hr, and then the OGTT was assessed after oral administration of 1 g/kg dextrose by gavage. At the indicated times, a drop of blood was collected from the tail vein to measure glucose concentration by the OneTouch® Ultra® Blue Test Strips, and additional 50–70 μl of blood was collected in Microvette 100 Li Heparin (Sarstedt). Blood collected was centrifuged at 4°C for 10 min at 9,330 g. The plasma supernatant was collected and used to measure serum insulin with the STELLUX® Chemi Rodent Insulin ELISA kit (Alpco).

### 2.5 Statistical analysis

Data were analyzed using Graphpad Prism 6 for Mac OS X (v6.0h). One‐way ANOVA was followed by Tukey post‐test to compare means between groups. Prior to averaging of replicates, outliers were removed by unbiased ROUT (robust regression followed by outlier identification) analysis at high stringency (Q = 0.01%). Averages of replicates for exocytosis and insulin secretion data for each donor are shown in Table . Correlations were initially assessed by generating a Pearson's correlation matrix and calculating two‐tailed P‐values, and this was followed by linear regression analysis (95% confidence intervals are shown). Data presented as scatterplots show the mean ± standard error. *p*‐values of < .05 were taken as statistically significant.

## 3 RESULTS

### 3.1 Variation in human islet responses to Exendin 4

**Figure 1:**
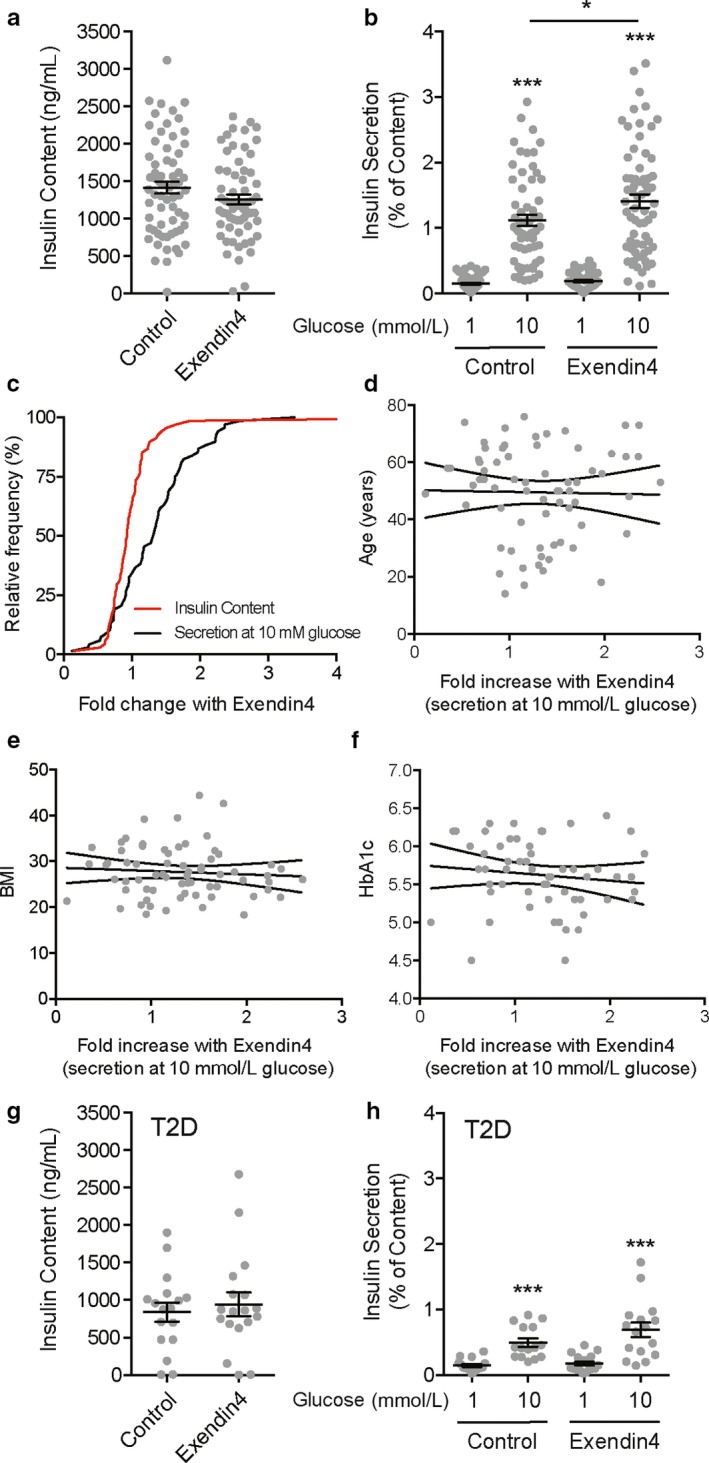
Exendin 4‐induced insulin secretion from isolated human islets is variable and modest***.*** (a) Insulin content measured from islets of donors without diabetes is not affected by acute (2 hr) treatment with 10 nM Exendin 4. (b) Glucose‐stimulated insulin secretion, as a percentage of content, from these islets is significantly but modestly increased by 10 nM Exendin 4. (c) The cumulative frequency distribution of fold‐changes in each donor to 10 nM Exendin 4 is shown for insulin content (red, a parameter that is unaffected by the 2 hr Exendin 4 treatment) and insulin secretion at 10 mM glucose (black). (d–f) Correlation analysis by linear regression shows no association between responsiveness to Exendin 4 and donor age (d), BMI (e), or HbA1c (f). Not shown, we also observe no association with donor sex, time in hospital, hospital blood glucose measures, organ cold ischemia time, or islet culture time post isolation. (g‐h) Similar to panels A and B, insulin content and secretion data from islets of donors with type 2 diabetes (T2D) with vehicle or 10 nM Exendin 4. Data are from 68 donors without diabetes and 18 donors with T2D. *‐*p* < .05 and ***‐*p* < .001 versus 1 mM glucose or as indicated

We measured glucose‐stimulated insulin secretion from 86 human islet preparations in the presence and absence of 10 nM Exendin 4 (Figure 1). In the islet preparations from donors without diabetes (Figure 1a‐f) the insulin content of groups treated acutely with Exendin 4 during the static secretion experiment was unaffected (Figure 1a). Insulin secretion was increased significantly in the presence of 10 mM glucose (*p* < .05), however, this was modest and on average we find that human islet preparations show only a 30.3 ± 6.6% (*n* = 68; Figure 1b) increase in insulin secretion with 10 nM Exendin 4. The maximum response that we observed was a 159% increase in secretion elicited by Exendin 4. Plotting the cumulative distribution of responses of individual donors demonstrates an appreciable rightward shift in the secretory response at 10 mM glucose upon Exendin 4 treatment (the fold‐change of insulin content upon acute Exendin 4 exposure is shown for comparison; Figure 1c). The modest and variable response of isolated human islets to Exendin 4 prompted us to examine donor or preparation factors that may explain these responses. However, we find no appreciable correlation between secretory responses to Exendin 4 and various parameters that include the age, BMI, or HbA1c of donors without diabetes (Figure 1d‐f) or several other factors (not shown: days in hospital, gender, blood glucose level in hospital, organ cold ischemia time, islet culture time). Finally, in islets from donors with type 2 diabetes we find similarly that acute exposure to Exendin 4 has no effect on insulin content (Figure 1g) and modestly increases insulin secretion at 10 mM glucose, although this did not reach statistical significance (Figure 1h).

### 3.2 Possible impact of an exocytosis amplifying pathway on Exendin 4 responsiveness

**Figure 2:**
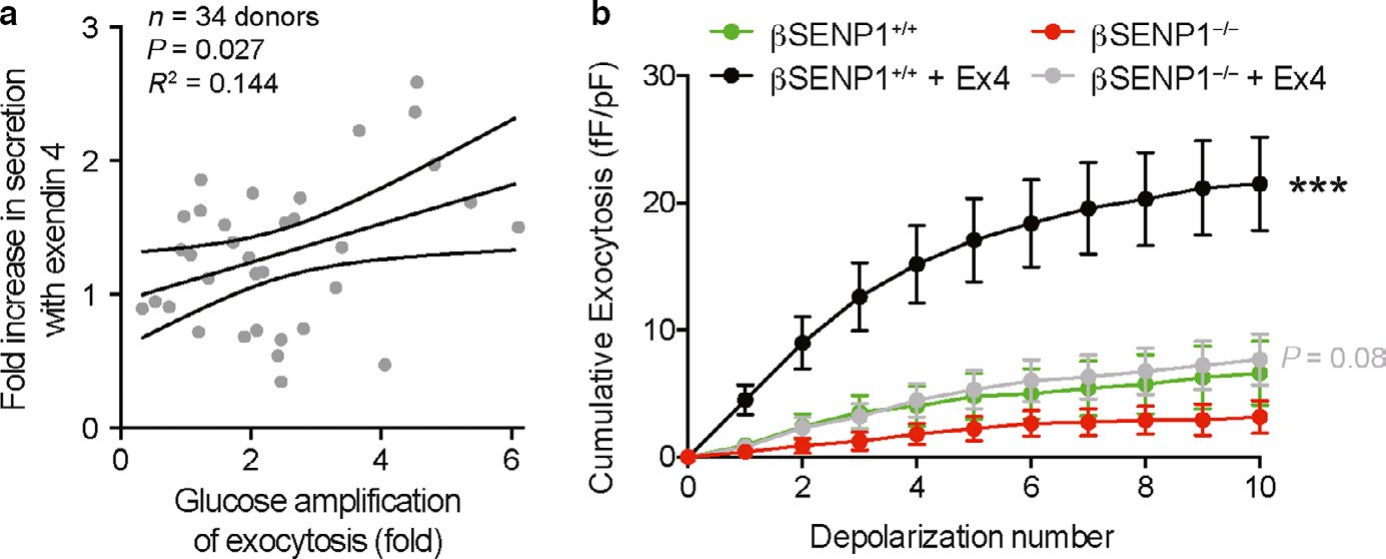
Relationship of Exendin 4 responses to glucose‐dependent amplification of β‐cell exocytosis*.* (a) Linear regression demonstrates a weak, but positive, correlation between the ability of Exendin 4 to enhance insulin secretion and glucose‐induced facilitation of exocytosis at the single β‐cell. Data are from 28 donors with exocytosis measured from at least three β‐cells. (b) Cumulative exocytosis from β‐cells (confirmed by insulin immunostaining) subjected to a series of 10 depolarizing pulses. Exocytosis is facilitated by 100 nM Exendin 4 in cells from βSENP^+/+^ mice, but not βSENP^−/−^ mice. Data are from 24–37 cells for each group, from four βSENP^+/+^ and four βSENP^−/−^ mice. ***‐*p* < .001 compared with WT vehicle control; in grey is the *p*‐value comparing Exendin 4 in the knockout with its vehicle control (red)

Glucose increases insulin secretion in part by facilitating the ability of insulin granules to undergo exocytosis in response to a Ca^2+^‐trigger (Dai et al., 2011; Ferdaoussi et al., 2015). In many of our human islet preparations we measure the glucose‐dependent amplification of insulin secretion by patch‐clamp electrophysiology. While we found no donor/isolation parameters that correlated with responsiveness of human islet preparations to Exendin 4, a modest, but significant, correlation was observed between the ability of glucose to facilitate β‐cell exocytosis (calculated as the fold increase in depolarization‐induced exocytosis at 10 vs. 1 mM glucose) and the ability of Exendin 4 to enhance insulin secretion at 10 mM glucose (Figure 2a). This suggests that the responsiveness of human islets to Exendin 4 may depend on the extent to which glucose is acting to ‘prime’ granules for Ca^2+^‐induced exocytosis. We showed that the deSUMOylating enzyme SENP1 is required for glucose‐dependent facilitation of insulin exocytosis (Ferdaoussi et al., 2015), likely by acting on proteins at the exocytotic site (Dai et al., 2011; Ferdaoussi et al., 2017). To test whether the SENP1‐dependent amplification pathway is required for Exendin 4‐mediated effects on insulin exocytosis, we measured exocytosis in mouse β‐cells lacking SENP1. Indeed, loss of SENP1 abolishes the exocytotic response to direct depolarization of β‐cells (Figure 2b).

### 3.3 β‐cell SENP1 is required for improvement of glucose tolerance with a DPPIV inhibitor

**Figure 3:**
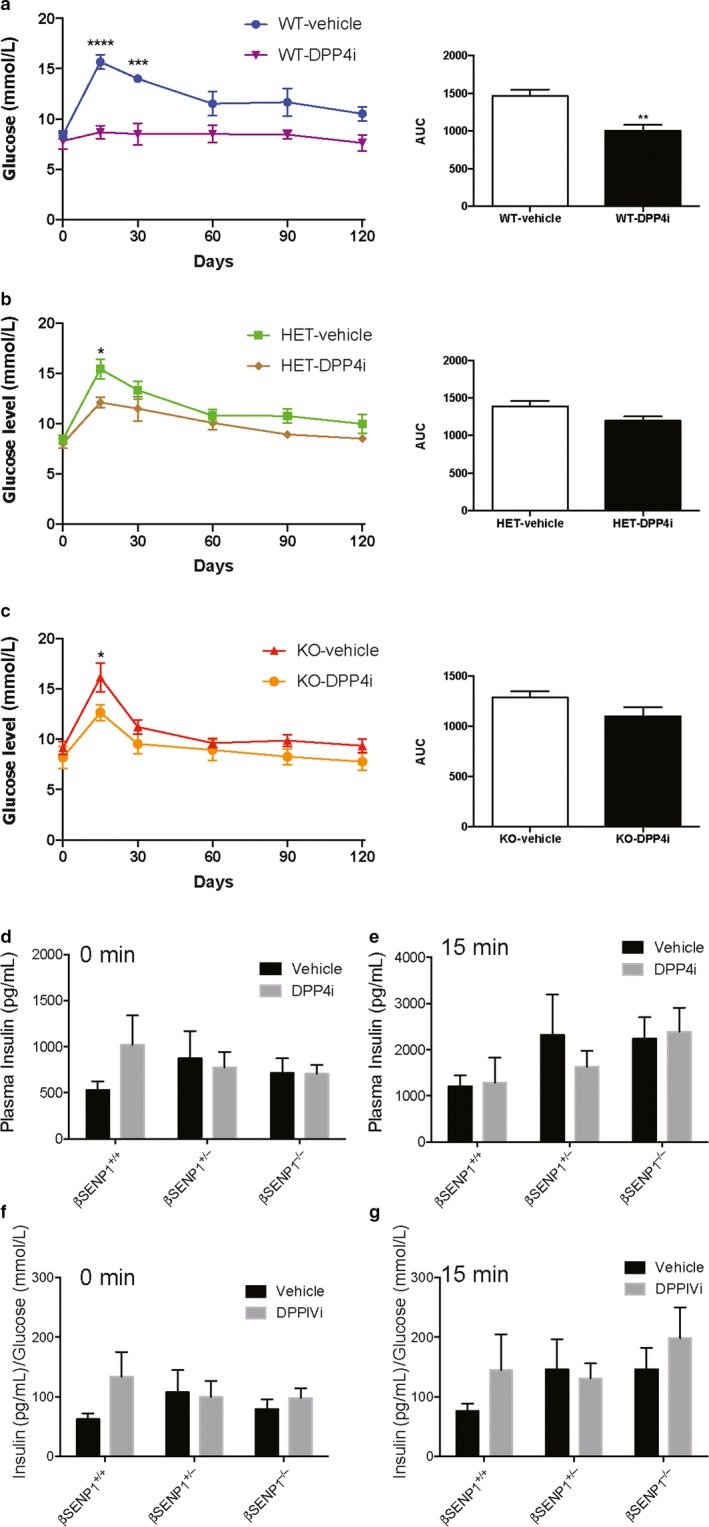
Acute improvement in glucose tolerance depends on β‐cell SENP1. Oral glucose tolerance, and the area under the curve (AUC) of glucose responses in male littermate βSENP^+/+^ (a; WT; *n* = 4, 4 mice), βSENP^+/−^ (b; HET; *n* = 6, 5 mice), and βSENP^−^^/^^−^ (c; KO; *n* = 8, 7 mice) mice at 1 hr following gavage of the DPPIV inhibitor MK‐0626 (3 mg/Kg). Loss of β‐cell SENP1 blunts the ability of DPPIV inhibition to improve glucose tolerance. Plasma insulin responses are shown at the 0 min (d) and 15 min (e) time points, and also normalized to measured glucose levels at these points (f,g). *‐*p* < .05, **‐*p* < .01, and ***‐*p* < .001 comparing vehicle control versus DPPIV inhibitor

The above data suggest that the responsiveness of islets to therapeutic approaches that increase incretin signaling might require intact metabolic signaling via SENP1. We therefore examined glucose tolerance and in vivo insulin responses to a DPPIV inhibitor MK‐0626 given either acutely 1 hr before an oral glucose‐tolerance test, or after 4 weeks of daily treatment. Upon acute DPPIV inhibitor treatment oral glucose tolerance was significantly improved in male wild‐type mice (βSENP1^+/+^) (Figure 3a). Mice that were either heterozygous (Figure 3b) or homozygous (Figure 3c) for β‐cell SENP1 knockout (βSENP1^+/−^ or βSENP1^−^^/^^−^) showed only minimal improvement in glucose tolerance in response to acute DPPIV inhibitor treatment. Plasma insulin tend to increase in βSENP1^+/+^ mice following DPPIV inhibitor treatment but not in the βSENP1^+/−^ or βSENP1^−^^/^^−^ mice (Figure 3d,e), and this was more obvious when shown as a ratio of plasma insulin/ glucose (Figure 3f,g), but did not reach statistical significance by two‐way ANOVA.

**Figure 4:**
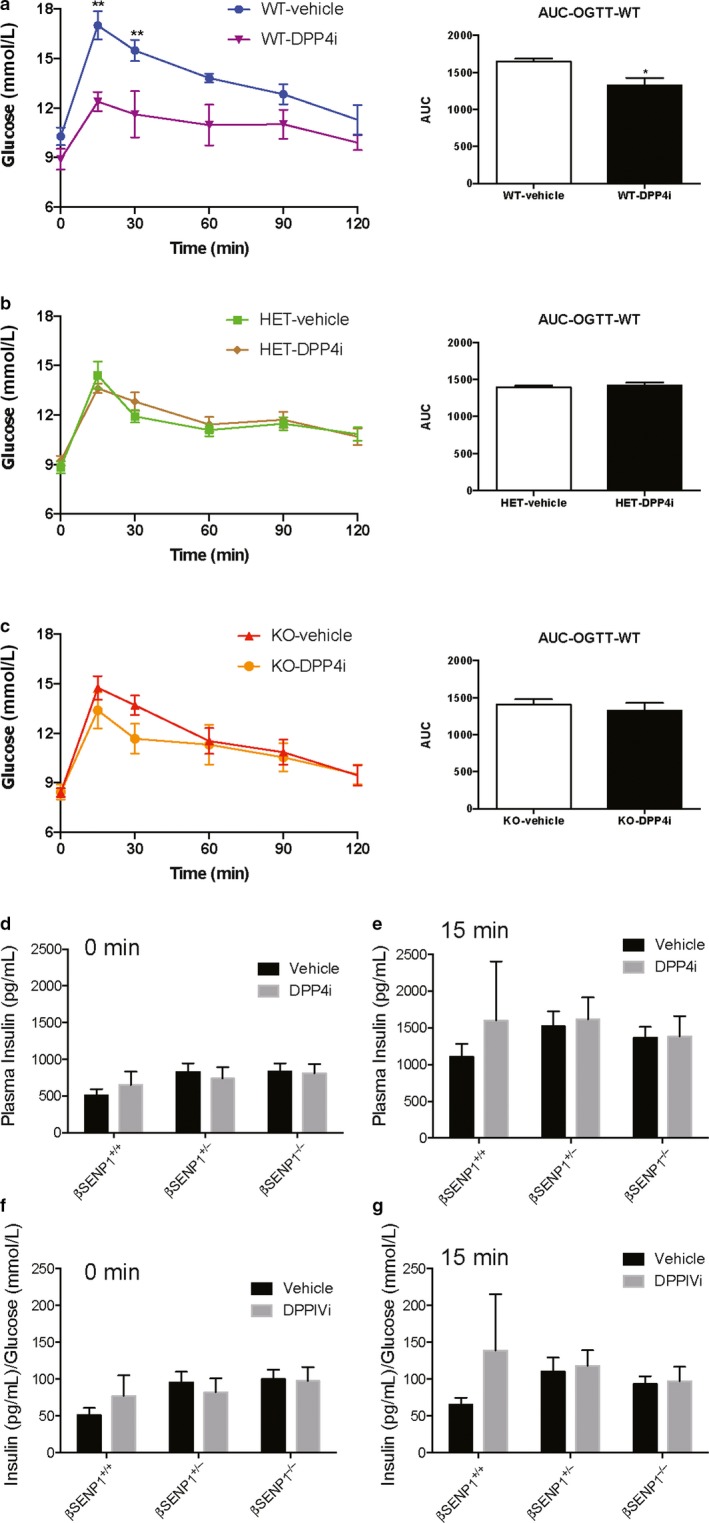
Chronic improvement in glucose tolerance depends on β‐cell SENP1. Oral glucose tolerance, and the area under the curve (AUC) of glucose responses in male littermate βSENP^+/+^ (a; WT; *n* = 5, 5 mice), βSENP^+/−^ (b; HET; *n* = 7, 7 mice), and βSENP^−/−^ (c; KO; *n* = 8, 8 mice) mice following 4 weeks of DPPIV inhibitor (MK‐0626, 3 mg/Kg) daily by gavage. Loss of β‐cell SENP1 blunts the ability of DPPIV inhibition to improve glucose tolerance in this setting. Plasma insulin responses are shown at the 0 min (d) and 15 min (e) time points, and also normalized to measured glucose levels at these points (f,g). *‐*p* < .05, and **‐*p* < .01 comparing vehicle control versus DPPIV inhibitor

Similar results were observed following 4 week treatment (and then overnight washout) with DPPIV inhibitor (Figure 4). Glucose tolerance was improved in β‐SENP1^+/+^ mice (Figure 4a), but not in littermate βSENP1^+/−^ (Figure 4b) or β‐SENP1^−^^/^^−^ (Figure 4c) mice. Again, plasma insulin levels tended to be increased in the wild‐type mice (Figure 4d,e), but not the βSENP1^+/−^ or βSENP1^−^^/^^−^ mice and was more obvious when compared with circulating glucose levels (Figure 4f,g), but did not reach statistical significance by ANOVA.

## 4 DISCUSSION

Here we showed that a key pathway linking β‐cell glucose metabolism to the insulin‐granule fusion site is required for incretin‐mediated facilitation of insulin exocytosis and responsiveness to DPPIV inhibition in vivo. While it is largely assumed that the glucose‐dependence of incretin/DPPIV inhibitor actions results from an inability to stimulate secretion in the absence of a glucose‐dependent Ca^2+^ response, the data here suggests an alternative or complimentary mechanism. Namely, an enhancement of insulin secretion by GLP‐1‐receptor activation requires the ‘priming’ of insulin granules by a metabolic amplification pathway mediated by the deSUMOylation enzyme SENP1.

We find that the responsiveness of human islet preparations to Exendin 4 is variable. In our hands some preparations fail completely to respond to Exendin 4, and this does not correlate with the ability of these preparations to respond to glucose – since many of these have excellent glucose‐induced insulin secretion (not shown). While the presence of nonresponding islet preparation fits with known patients that are ‘nonresponders’ to incretin mimetic or DPPIV inhibitor therapies (Kanamori and Matsuba, 2013; Jones et al., 2014), it remains undetermined whether the two findings are linked (i.e. whether ‘non‐responding’ patients have ‘nonresponding’ islets). Genetic contributions to incretin responsiveness may also play a role and have not been investigated in the present study. Somewhat more perplexing than variation in secretory responses to Exendin 4 (which might have been expected), is the overall poor responsiveness of these islets to Exendin 4 in vitro (an average 30% increase in insulin secretion). This is less than expected based on recent rodent islet studies, but generally consistent with the limited number of human islet studies that have been published using similar static incubation methods (e.g. Kolic et al., 2014). The reason for this low responsiveness to GLP‐1 receptor activation is not entirely clear, but several possible contributing factors could be explored: the possible isolation/stress induced production of GLP‐1 by α‐cells in these preparations could already saturate GLP‐1 receptor signaling (Chambers et al., 2017); a loss of islet innervation (Rodriguez‐Diaz et al., 2011); or loss of islet vasculature which may regulate islet blood flow in response to manipulation of incretin signaling (Samikannu et al., 2013). We should note also that the concentration of Exendin 4 used here (10 nM), while within the range often used experimentally, is higher than the picomolar concentrations normally achieved in patients (Cirinclone and Mager, 2017).

The ability of Exendin 4 and DPPIV inhibitor to increase insulin secretion (and improve glucose tolerance) appears to depend on the expression of SENP1 within β‐cells, as indicated both by the inability of Exendin 4 to enhance β‐cell exocytosis in the βSENP1^−/−^ cells and the inability of the MK‐0626 DPPIV inhibitor to increase plasma insulin acutely and improve glucose tolerance in either the βSENP1^+/−^ or the βSENP1^−^^/^^−^ mice. SENP1 is an isopeptidase that selectively cleaves SUMO peptides from their target proteins (Kunz, Piller, and Müller, 2018). SENP1 is redox‐sensitive (Xu et al., 2008) and within β‐cells appears to be activated downstream of the glucose‐induced mitochondrial export of (iso)citrate (Ferdaoussi et al., 2015). In this scheme, the resulting generation of NADPH in the cytosol, coupled to regeneration of reduced glutathione, increases SENP1 catalytic activity via the action of glutaredoxin, which itself has been shown to control β‐cell exocytosis (Ivarsson et al., 2005; Reinbothe et al., 2009). Downstream targets include synaptotagmin VII and tomosyn (Dai et al., 2011; Ferdaoussi et al., 2017) in the β‐cell but may also include other exocytotic proteins that have now also been shown to be controlled by SUMOylation (Ferdaoussi and MacDonald, 2017). In the present context it is interesting that at least some of these SENP1 target proteins overlap with phosphorylation targets downstream of the GLP‐1 receptor. Chief among these is the exocytotic Ca^2+^ sensor synaptotagmin VII, the phosphorylation of which is thought to be important for incretin‐induced insulin secretion (Wu et al., 2015). This provides an interesting potential mechanism where by the glucose‐dependence of GLP‐1 receptor action on insulin secretion results from an overlap of SENP1 and PKA (and maybe EPAC) targets controlling secretion.

## CONFLICT OF INTEREST

This work was funded in part by a grant from Merck. The authors have no other financial or material conflicts to report.

## Supporting information



Table S1.
